# Chemical composition and antibacterial properties of *Peganum harmala* L.

**DOI:** 10.22038/AJP.2019.13382

**Published:** 2019

**Authors:** Milad Iranshahy, Sedigheh Fazly Bazzaz, Giti Haririzadeh, Bibi Zahra Abootorabi, Ali Mohammad Mohamadi, Zahra Khashyarmanesh

**Affiliations:** 1 *Department of Pharmacognosy, School of Pharmacy, Mashhad University of Medical Sciences, Mashhad, Iran*; 2 *Biotechnology Research Center, Pharmaceutical Technology Institute, Mashhad University of Medical Sciences, Mashhad, Iran*; 3 *Pharmaceutical Control Department, School of Pharmacy, Mashhad University of Medical Sciences, Mashhad, Iran*; 4 *Medicinal Chemistry Department, School of Pharmacy, Mashhad University of Medical Sciences, Mashhad, Iran*

**Keywords:** Alkaloid, Antimicrobial activity, Peganum harmala, Harmine, Harmaline, Smoke

## Abstract

**Objective:**

The present study was conducted to investigate antibacterial properties of fruit and flower of *Peganum harmala*.

**Material and Methods:**

Column chromatography, followed by preparative thin layer chromatography (TLC) was used for final purification. The structure of pure alkaloids was determined using spectroscopic methods (^1^H-NMR, ^13^C-NMR, UV and MS). Smoke and extract of total alkaloids were investigated for antimicrobial activity against five different microorganisms (standards and hospital isolates). The antibacterial activity was evaluated using disc diffusion assay and minimum inhibitory concentration (MIC) was determined by serial dilution methods.

**Results:**

Chemical investigation of the chloroform extract of ripe fruit and flower of *P. harmala* led to identification of three alkaloids in ripe fruit and two alkaloids in the flower and leaves of this plant. Alkaloids identified in ripe fruit were harmine, peganine (vasicine) and harmaline. Two alkaloids, harmine and peganine, were detected in the flower of *P. harmala, *while harmaline was only found in the ripe fruit. The total alkaloids of flower were compared with total alkaloids of ripe fruit by TLC method. Fruits and flowers had 3.12 and 3.27% alkaloid contents, respectively.

**Conclusion:**

Our results showed that the alkaloids and smoke were specifically more effective on *Candida albicans* and Gram- positive bacteria (*Micrococcus luteus* and *Staphylococcus aureus*), while Gram- negative bacteria, especially *Pseudomonas aeruginosa*, were less sensitive.

## Introduction

*Peganum harmala* L. (Zygophyllaceae) is a herb native to dry area from east Mediterranean to northern India and is widely spread in Iran (Branch, 2012). It is claimed that the plant has been used as an important medicinal plant in global folk medicine (Sharaf et al., 1997); also, burning its seeds was used in Iran as an antiseptic and disinfectant approach (Ghasemi Pirbalouti et al., 2013). While consuming the seeds can stimulate the central nervous system (CNS), can cause paralysis and is poisonous at high doses, the smoke of *P. harmala* seeds is used as an antimicrobial approach in folk medicine (Al-Shamma et al., 1981; Achour et al., 2012; Moloudizargari et al., 2013; Lamchouri, 2014; Mina et al., 2015).

*P. harmala* has various alkaloids of diverse structures that are difficult to be synthesized; however, the extract from its ripe fruit or flower contains structurally easier compounds.* P. harmala *contains β-carboline and quinazoline alkaloids which are responsible for the toxicological and pharmacological effects of the plant. β- carbolin alkaloids are abundant in many plant families and possess neurostimulant and monoamine oxidase (MAO) inhibitory activities (Herraiz et al., 2010).

Moreover, antihypertensive, hallucinogenic, and antidepressant effects have been reported for *P. harmala* (Moloudizargari et al., 2013). Traditionally, *P. harmala* has been used to treat various ailments including Parkinson's disease, gastrointestinal and cardiovascular disorders and certain types of malignancies (Moloudizargari et al., 2013).  More recently, the antileishmanial activities of harmine were studied in detail (Mirzaie et al., 2007). Splettstoeser et al. reported that *P. harmala* alkaloids have protective effects on neurons against the excitotoxicity of dopamine and glutamate (Splettstoesser et al., 2005).

β-Carbolin alkaloids attracted the scientists' attention because of their potent inhibitory effects against certain antibiotic-resistant strains (Suzuki et al., 2018). In this article, we studied the antimicrobial activity of alkaloid extract and smoke of *P. harmala* against standard and hospital isolates.

## Materials and Methods

***P. harmala*** **Collection and Extraction**

*P. harmala* was collected from Neishabur (Khorasan Razavi Province) in July, 2017. The dried and powdered herb (3 kg) was macerated four times with 3 l of methanol for 24 hr. The extracts were combined and the solvent was evaporated to dryness, using a rotary evaporator. The residue was kept at -20°C until used. 

**Preliminary phytochemical screening**

The qualitative analysis of the chemical constituents was carried out based on the methods previously described by Farnsworth (1996) with some modifications (Farnswort, 1996; Siddiqui et al., 1987; Kaskoos, 2014).

**Alkaloids determination**

Concentrated methanol extract (3g) was suspended in H_2_SO_4_ (3%) for 3 hr, stirred and filtrated. Then, it was basified using 10% NH_4_OH (pH 8-9) and extracted by CHCl_3_ (three times). Then, the extract was concentrated using rotary evaporator under vacuum, and spotted on a thin layer chromatography (TLC) plate. After development, the plate was dried, and sprayed with Dragendorff's reagent and orange spots were considered the alkaloids.

**Saponins determination**

To 1 g of methanol extract, 10 ml of water was added and the mixture was shaken vigorously for two minutes and after 30 min, the formation of a rich lather (which is stable for more than 10 min) was investigated.

**Flavonoids determination**

Metallic magnesium (100 g) was added to 1 g methanol extract in 10 ml water, and then, 5-6 drops of concentrated hydrochloride acid (HCl) were added slowly and dropwise. After adding 2 ml of amyl alcohol, the solution turns red if flavonoids are present in the extract.

**Tannins determination**

Two ml of 10% methanol extract was added to 0.1 g gelatin, 10 ml water and 1g sodium chloride. Precipitation is indicative of the presence of tannins.

**Cyanogenetic glycosides determination**

For qualitative detection of cyanogenetic glycosides in the plant, the Grignard test was employed. Briefly, 4 drops of toluene were added to about 2 g of moist plant material in a small test tube. 

The Grignard reagent (a solution of 0.5 g picric acid, 5 g Na_2_CO_3_, and water q.s. 100 ml) was used as the indicator. The strips of filter paper were saturated with the solution, dried and introduced into the neck of the test tube containing the plant material. The test tube and contents were then warmed at 30-35^ο^C for up to 3 hr. Any change in color of the yellow test paper to red, is indicative of cyanogenetic glycosides while absence of a red color after 3 hr is considered a negative result.

**General experimental procedures**

UV, NMR and mass spectra were recorded on Shimadzu UV1650 spectrometer, Bruker 100 MHz NMR spectrometer (Bruker, Germany) and Bruker Mass spectrometer (Bruker, Germany), respectively.

**Extraction of alkaloids**

The residue (obtained as was described in the extraction part) was acidified using 3% sulfuric acid (pH 1), filtered and washed with petroleum ether to remove high lipophilic compounds. The aqueous acid layer was basified to pH 8-9 using NH_4_OH (10%) and extracted several times using chloroform. The chloroform layer was mixed and the solvent was evaporated to dryness. Extraction of alkaloids was done by column chromatography with silica gel. For 1 g of the total alkaloid, 50 g silica gel (G60, mesh 230) was used. The polarity of the solvent system gradually increased from chloroform-methanol 95-5% to methanol 100%.

The collected fractions were tested for the presence of alkaloid by TLC using different solvent systems (chloroform/methanol/ammonia) and Dragendorff's reagent as indicator. 

Final purification of alkaloids was done by preparative TLC and the structure of the purified compounds was elucidated using ^1^H-NMR, ^13^C-NMR, UV and MS (supporting information).

**Antimicrobial effects of alkaloids and smoke**

**Preparation of alcoholic extract of alkaloids**

The seeds of the plant were washed with petroleum ether, dried and ground into crude powder. In the first step, 100-150 g of dried powder was macerated in 1 L of 95% methanol for 12 hr at 50°C in water bath. After evaporating the solvent (i.e. methanol), the residue was dissolved in HCl (5%) until the pH of the final solution became 1, then the solution was filtered.

In the next step, the filtrate was extracted twice by 30 ml petroleum ether to remove highly lipophilic compounds. The residue was basified by 10% NH_4_OH (pH 9-10) and extracted by chloroform (30 ml); finally, the chloroform was evaporated to dryness.

**Tested microorganisms**

*Micrococcus luteus *(PTCC 9341),* Escherichia coli* (ATCC 8739), *Pseudomonas aeruginosa* (PTCC 1074) *Staphylococcus aureus* (ATCC 29737), and the yeast *Candida albicans* (ATCC 10231) were provided as lyophilized by Persian Type Culture Collection, Tehran, Iran. The pathogenic strains (hospital strains including one strain from* E. coli*, *P. aeruginosa,* *S. aureus* and *C. albicans*) were provided by University Hospital of Imam Reza, Mashhad, Iran.

**Antimicrobial evaluations**

**Revival of microorganisms**

Soybean-casein digest broth medium (0.5 ml) was added to lyophilized microorganisms and the suspension was used for preparation of master culture (soybean-casein digest agar) by streaking method. The culture medium was incubated (24 hr, at 37^ο^C for bacteria and 48 hr, at 25^ο^C for yeast) and then, the sub-master culture was prepared. From these sub-master cultures, using normal saline, the microbial count of 10^8^ CFU/ml (comparing with 0.5 ml McFarland standard) was prepared. 

**Antimicrobial properties of total alkaloids**

Alkaloids extract was added to the culture media (Mueller Hinton agar, 45^ο^C) to prepare different concentrations of alkaloids (20-3000 µg/ml). The standard antibiotic powders, amikacin (from 0.25-100 µg/ml) and clotrimazole (from 0.25-20 µg/ml) were also prepared in the same manner. To inoculate each microorganism, 15 ml of culture medium was transferred to the plate and 0.5 ml from 10^5^ CFU/ml suspension of microorganism was spread on the surface of the agar plate. Incubation of plates was performed at 37^ο^C for 24 hr for bacteria and at 25^ο^C for 48 hr for yeast; finally, the growth of microorganisms was investigated.

**Smoke**

**Figure 1:**
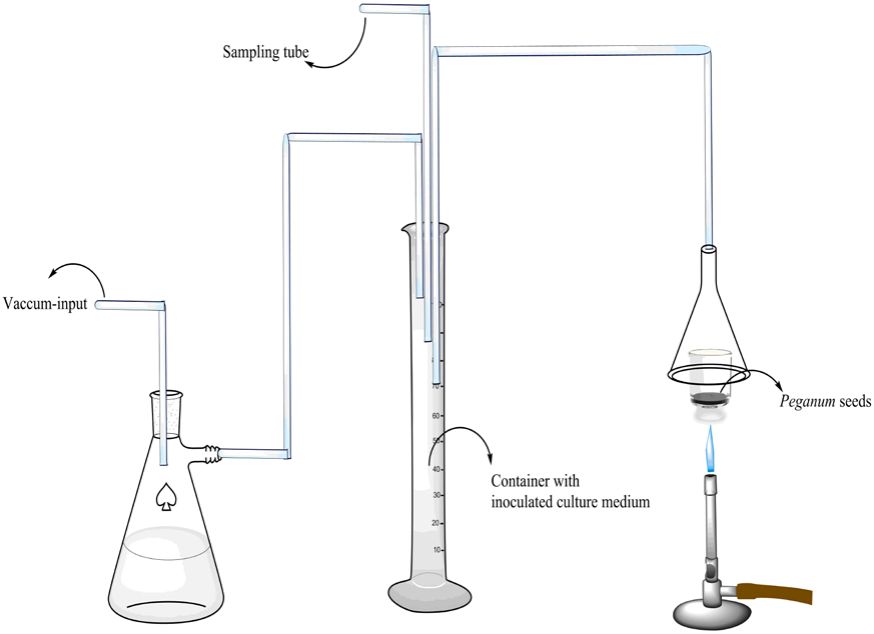
A schematic view of the apparatus used in this study for investigation of the antibacterial activity of *P. harmala *seeds

The smoke of burned seeds of *P. harmala* or formalin tablet in a watch glass was directed to a flask containing Mueller Hinton broth (150 ml) through a tube. One ml of each microbial suspension (equivalent to 0.5 ml McFarland standard, i.e. 10^8^ CFU/ml) was added to the medium (Figure 1). Incubation of the flask was performed for 24 hr, at 37^ο^C for bacteria and for 48 hr, 25^ο^C for yeast and then, the growth of microorganisms was observed. All the steps were performed under aseptic conditions.

## Results

**Phytochemical screening**

The results of phytochemical screening of flowers and ripe fruit from *P. harmala *extracts are shown in Table 1. The phytochemical analysis was carried out to detect the presence of phytoconstituents (alkaloids, tannins, saponins and flavonoids) in methanol extracts of *P. harmala* which indicated the presence of saponins and alkaloids.

**Table 1:** Phytochemical screening results

**Plant Part**	**Cyanogenic glycosides**	**Saponin**	**Tannin**	**Flavonoid**	**Alkaloid**
**Flower and leaves **	_	+	_	_	++++
**Ripe fruit and leaves **	_	+++	_	_	++++

**Antimicrobial activity**

Minimum inhibitory concentration (MIC) of total alkaloids against standard microorganisms *M. luteus, E. coli, P.* *aeruginosa *and *S. aureus* was 31.25±1.65 µg/ml, 500±81.60 µg/ml, 1500±40.82 µg/ml and 125±2.04 µg/ml, respectively. MIC values for hospital isolates were 1500±16.32 µg/ml*, *2000±40.82 µg/ml and 150±4.08 µg/ml for* E. coli*, *P. aeruginosa*, and *S*. *aureus*, respectively. In case of *C. albicans*, MIC was 62.5±2 µg/ml for both standard and hospital isolate (Table 2). The MIC test for each standard and hospital strain was performed in triplicate and the results are expressed as mean±standard deviation.

**Table 2:** MICs (µg/ml) of total alkaloid extract, clotrimazole and amikacin against standard and pathogen microorganisms

**Microorganism**	**Standard** **(total alkaloid) **	**Pathogen** **(total alkaloid) **	**Standard and pathogen** **(clotrimazole)**	**Standard** **(Amikacin) **	**Pathogen** **(Amikacin) **
***Staphylococcus aureus***	125±2.04	150±4.08	-	8±0.163	15±0.408
***Escherichia coli***	500±8.160	1500±16.32	-	4±0.408	10±0.816
***Pseudomonas aeruginosa***	1500±40.820	2000±40.82	-	4±0.408	50±1.632
***Candida albicans***	62.5±2.04	62.5±2.04	8±0.204	-	-
***Micrococcus luteus***	31.25±1.650		-	4±0.408	-

The MIC of *P. harmala* smoke against standard microorganisms* M. luteus*, *E. coli, P. aeruginosa, S. aureus *and *C. albicans* was 2.25±0.04, 1±0.082, 6±0.122, 4±0.82 and 1±0.04 (smoke equivalent g of seed), respectively. The MIC for hospital isolates was 2±0.224, 8±0.408, 5±0.163, and 1±0.04 against* E. coli*, *P. aeruginosa*, *S. aureus *and *C. albicans*, respectively (smoke equivalent g of seed) (Table 3).

**Table 3:** Antimicrobial activity (equivalent of g seeds) of smoke against standard and pathogenic microorganisms

**Microorganism (standard)**	**Standard** **(smoke)**	**Pathogen** **(smoke)**	**Standard** **(Formalin tablets)**	**Pathogen** ** (Formalin tablets)**
***Staphylococcus aureus***	4±0.082	5±0.163	2±0.204	2±0.204
***Escherichia coli***	1±0.082	2±0.224	0.5±0.144	1.5±0.204
***Pseudomonas aeruginosa***	6±0.122	8±0.408	0.5±0.144	2±0.204
***Candida albicans***	1±0.04	1±0.04	1±0.163	1±0.163
***Micrococcus luteus***	2.25±0.04		0.5±0.144	-

Amikacin was the positive control for evaluation of antibacterial activity of total alkaloids and its MIC against standard microorganisms*, M. luteus, E. coli, P. aeruginosa *and *S. aureus* was 4±0.408 µg/ml, 4±0.408 µg/ml, 4±0.408 µg/ml and 8±0.163µg/ml, respectively. MIC of amikacin for hospital isolates was 15±0.408 µg/ml, 10±0.816 µg/ml and 50±1.632 µ/ml for *S. aureus, E. coli* and *P. aeruginosa*, respectively (Table 2).

Formalin was used as positive control for evaluation of the antimicrobial activity of smoke and its MIC against standard microorganisms*, M. luteus, E. coli, P. aeruginosa, S. aureus* and *C. albicans* was 0.5±0.144, 0.5±0.144, 0.5±0.144, 2±0.204 and 1±0.163 (Number of formalin pills), respectively. For hospital isolates, formalin MIC was 2±0.204*, *1.5±0.204*, *2±0.204 *and* 1±0.163 (Number of formalin 1000 mg pills, Table 3) for *S. aureus, E. coli, P. aeruginosa* and *C. albicans*, respectively. 

**Characterization of alkaloids**

**Figure 2:**
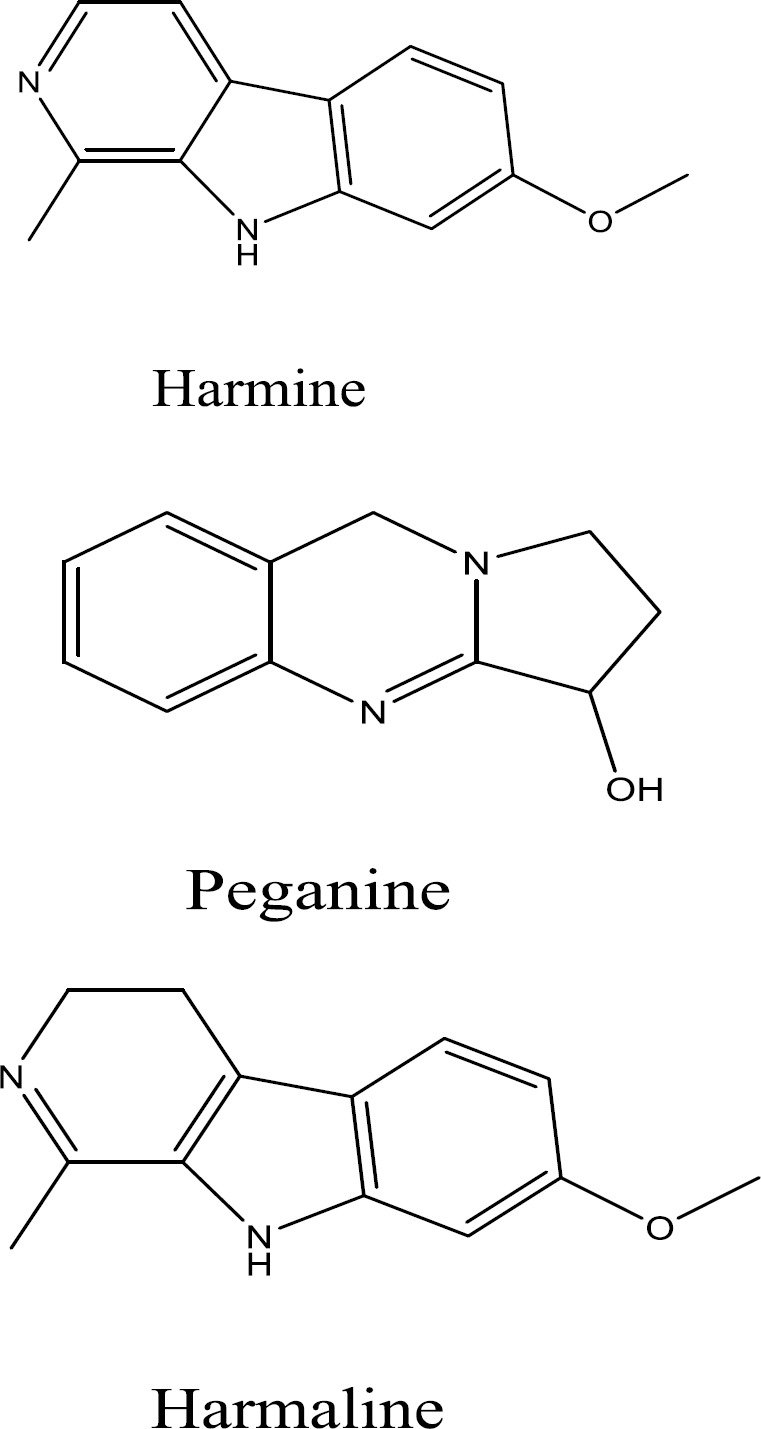
Chemical structures of alkaloids isolated from *P. harmala*

Three known alkaloids, namely harmine, peganine and harmaline (Figure 2) were isolated from *P. harmala* and their structures were confirmed via comparing ^1^H-NMR, ^13^C-NMR, UV and MS spectra with those reported in the literature (Manske, 1954; Chatterjee and Ganguly, 1968; Brossi, 1985; Siddiqui et al., 1988; Ayoub et al., 1989). For detailed information on structure elucidation of the alkaloids, please see the data presented in supporting information.

## Discussion

For many years, medicinal plants have been considered a potential source of pharmaceutical natural products. *Peganum harmala* is one of the plants that has been extensively used in traditional medicine of Iran and various parts of this plant including its seeds, bark, and root have been used for treatment of different ailments (Mina et al., 2015).

In this paper, phytochemical screening of the ripe fruit and flowers of *P. harmala*, was done. A summary of the results of the qualitative phytochemical analysis is shown in Table 1.

Our results showed that the content of total alkaloids in seeds and flowers was 3.12 and 3.27% respectively. So far, several studies have been done to extract and identify the alkaloids of this plant and most of these studies reported that the seed of the plant has the highest amount of alkaloids.

In 2012, Asgarpanah et al. reported that total alkaloid content of *P. harmala* varied between 2 and 5% which were consistent with our findings (Asgarpanah and Ramezanloo, 2012).

Herraiz et al. in 2010, reported that the highest levels of alkaloids are found in the seeds and roots followed by stems and leaves, but flowers lack such compounds (Herraiz et al., 2010). 

Bukhari in 2008 reported that in the *P. harmala*, the harman alkaloids (harmine and harmaline) are specifically located in the seeds (Bukhari et al., 2008). Hemmateenejad et al. in 2006 reported an analysis of *P. harmala* seeds and the results indicated 1.84, 0.16, 0.25 and 3.90% content for harmine, harmane, harmaline and harmalol, respectively (Hemmateenejad et al., 2006).

The number of alkaloids in *P. harmala* ripe fruit and flower extract in our study, was limited. It is generally accepted that the geographical location and plant growth conditions as well as the conditions of plant gathering, affect the amount and type of plant compounds, so this finding may be due to such variations.

Thin layer chromatography only indicated three alkaloids with very too close R_f_. The extracted alkaloids were polar, so the first solvent system used for column chromatography was chloroform- methanol (95:5). The polarity of the solvent gradually increased and fractions were purified using thin layer chromatography. The most suitable solvent system was chloroform- methanol-ammonia (60:30:0.5).

Thin layer chromatography showed that alkaloids in flower, leaves and ripe fruit are similar. These two alkaloids were harmine and peganine. Harmaline was the alkaloid that was only found in ripe fruit and did not exist in the flower of *P. harmala*. Spectroscopic properties of these alkaloids (assessed by UV, ^1^H-NMR, ^13^C-NMR and MS spectrum) are presented in supporting information.

The antibacterial activity of different parts of *P. harmala* has been investigated in many studies. Darabpour et al. in 2011 reported that the best antibacterial activity against Gram-positive bacterial species, including *Bacillus anthracis*, *Bacillus cereus*, *Bacillus pumilus*, *Staphylococcus aureus*, *Staphylococcus epidermidis*, *Listeria monocytogenes* and *Streptococcus pyogenes* and Gram-negative bacterial species, including *Pseudomonas aeruginosa*, *Brucella melitensis*, *Proteus mirabilis*, *Salmonella typhi*, *Escherichia coli* and *Klebsiella pneumoniae*, were observed for the seed and root extracts among the studied parts of *P. harmala*. Even at the lowest concentration, the root and seed extracts exhibited antibacterial activity against all of tested bacteria. The antibacterial effect of leaves was moderate while stem and flower extracts showed relatively poor activity (Darabpour et al., 2011). Other studies also revealed the inhibitory effect of seed alkaloid extracts of *P. harmala* against some Gram-positive bacterial strains such as *S. aureus* and *S. saprophyticus* and Gram-negative including *E. coli*, *K. pneumoniae*, *P. aeruginosa*, *Proteus mirabilis* and *Serratia* spp. The results indicated that the diameters of inhibition zones ranged from 11 to 22 mm for all treatments (Benbott et al., 2012). Also, aqueous and ethanol extracts of *P. harmala* were effective against all Gram-positive bacteria tested in a study including *Lactobacilli* and *Streptococcus* *mutans*, respectively (Cowan, 1999). Other studies revealed the sensitivity of *Aeromonas hydrophila* strain to seed aqueous extract of *P. harmala*, since inhibition zone was 20.5 (Abutbul et al., 2005).

In this work, we examined the activity of total alkaloids extract of seeds of *P. harmala* against five different microorganisms (standards and hospital strains). Our results showed that *P. harmala* total alkaloids have remarkably higher activity against Gram-positive bacteria than Gram-negative ones and the MIC against Gram-positive bacteria was less than MIC against Gram-negative bacteria. This differences in MIC could be due to the external membrane of Gram-negative bacteria that prevents the molecules from penetration to the cell.

Total alkaloids at 62.5 µg/ml concentration showed activity equivalent to 8 µg/ml clotrimazole against standard and pathogenic *C. albicans*. This suggests a good activity of alkaloids against this yeast. Even the activity of total alkaloids against this yeast was more marked than its activity against Gram-positive and Gram-negative bacteria (except *M. luteus*). The smoke of *P. harmala* also showed higher activity against *C. albicans* compared to other microorganisms tested, with the exception of *E. coli.*

According to our results, the order of antimicrobial activity of total alkaloids and the smoke of *P. harmala* was yeasts, Gram-positive bacteria and Gram-negative bacteria, respectively. 

Traditional medicine systems are still valuable sources of information to find biologically active plants and lead compounds for treatment of different diseases. In this study, the strong traditional background of *P. harmala* as a disinfectant, led us to unravel the antibacterial and especially antifungal activity of this plant. Future studies should mainly focus on isolation of compounds responsible for the observed activity and disclose the mechanism of action of such compounds. In addition, the results of this study propose the smoke of *P. harmala* seeds as a possible disinfectant that can be used safely for sanitary purposes. The chemical composition of the smoke of *P. harmala* seeds is another interesting topic remains to be elucidated in the future.
